# The impact of technological innovations on the environmental Kuznets curve: evidence from EU-27

**DOI:** 10.1007/s11356-024-32303-3

**Published:** 2024-02-17

**Authors:** Hamdi Ercan, Buket Savranlar, Melike Atay Polat, Yuksel Yıgıt, Alper Aslan

**Affiliations:** 1 Faculty of Aeronautics and Astronautics, Aviation Electrical and Electronics, Erciyes University, Kayseri, Turkey; 2 Vocational School, Accounting and Tax Applications, Nisantaşi University, Istanbul, Turkey; 3 Faculty of Economics and Administrative Sciences, Mardin Artuklu University, Mardin, Turkey; 4 Turkish Gendarmerie General Command, Ankara, Turkey; 5 Faculty of Aeronautics and Astronautics, Department of Aviation Management, Erciyes University, Kayseri, Turkey

**Keywords:** EKC, Income inequality, Innovations, EU, K32, O32, O15, C23

## Abstract

The EKC hypothesis expresses the inverted U-shaped relationship between per capita income and environmental quality. In the literature, the role of technological innovations and income inequality on pollution is a relatively recent discussion in the studies testing the EKC hypothesis. The aim of this paper is to investigate the impact of technological innovations, income inequality, exports, urbanization, and growth on CO_2_ emissions in EU-27. In addition, while investigating this relationship, exports and urbanization are also considered and panel vector autoregression (PVAR) analysis is applied for the 2005–2019 period. According to the coefficient estimation results, while income inequality, exports, and urbanization increase pollution, technological innovations contribute to environmental quality. Also, the results demonstrated that the EKC hypothesis is invalid in these countries and there is a U-shaped relationship between growth and emissions. The causality test results revealed the presence of unidirectional causality running from all explanatory variables to CO_2_ emissions. Moreover, impulse-response graphs demonstrated that the reply of emissions to the shocks in the explanatory variables is similar to the long-run coefficient results. In conclusion, all available empirical evidence for this relationship highlights that income inequality and technological innovations should be considered in policy-making processes to ensure environmental quality in EU-27 countries.

## Introduction

The use of fossil reserves such as coal and oil causes climate disorders and global warming by increasing carbon emissions, which contribute significantly to greenhouse gases (Zhu et al. 2018). Public interest in environmental problems arising from the common use of energy resources began in the 1960s. In this direction, the issue of environmental pollution has become debatable through economic growth, which many economic policies focus on (Shabani and Shahnazi 2019).

Economic development triggers industrialization and urbanization by encouraging the migration of people from rural areas to cities and shifting employment from the agricultural sector to the industrial sector (Wang et al. 2020a, b). Since energy consumption is the primary source of CO_2_ emissions, energy consumption and CO_2_ emissions are directly related to the process of industrialization and urbanization (Zi et al. 2016). The positive and significant impact of urbanization on CO_2_ emissions may lead to climate change policies being shaped within the scope of a CO_2_ emission reduction strategy (Sadorsky 2014). Therefore, many previous studies have investigated the relationship between urbanization and environmental quality.

Developing countries are between the lowest in terms of emissions compared to developed economies. This may be as a result of the low income, the level of economic growth, and differences in technological innovation and resource equipment. In summary, all countries contribute to emissions and therefore reducing emissions has become a global goal (Wu et al. 2021). Countries that try to improve environmental quality by slowing down global warming and reducing greenhouse gas emissions are trying to find a global solution to this problem by signing the Kyoto Protocol (Zhu et al. 2018). In contrast, the difference in emission targets has turned the issue of reducing carbon emissions into a problem in industrialized and emerging countries. This problem can be associated with the difference in income level and the difference in development stages. The hypothetical explanation of this relationship is supported by the environmental Kuznets curve (EKC) theory (Guo 2013). This theory has a significant place in the environmental economic literature in explaining the linkage between environment and economic development. The reversed U-formed connection for environmental deterioration and income is explained as the EKC hypothesis. The EKC is a hypothesis that the first stage of economic development causes environmental degradation, but it is associated with an improvement in environmental quality because of income levels exceeding a certain turning point (Heerink et al. 2001; Kasuga and Takaya 2017). As a result of the increase in income, the claim for environmental quality improvement and environmentally friendly investments increases. Because households’ consumption and production decisions such as agriculture have an impact on environmental quality, consumption and production patterns change as a result of increased household income (Heerink et al. 2001).

As the level of disparity affects the level and content of total consumption, environmental pressure is affected (Berthe and Elie 2015). Empiric papers based on the EKC hypothesis have mostly focused on CO_2_ emissions, which have a significant role in climate change as an indicator of pollution (Rojas-Vallejos and Lastuka 2020). While investigating the EKC hypothesis, income level is taken instead of income distribution as the determinant of environmental quality. However, in some academic papers, the issue of distribution in the link between environmental quality and income has also been taken into account (Coondoo and Dinda 2008). One of these papers belongs to Torras and Boyce (1998). Authors tested the nexus between different indicators of pollution and income inequality using regression analysis and found that higher income disparity was related to more contamination in low-income economies than in high-income economies. On the other hand, testing the hypothesis that political and economic inequality causes less environmental damage, Scruggs (1998) supported that distribution issues are insufficient to explain environmental quality.

Income disparity, which expresses that the income produced in a country and period is not evenly deployed among social classes, individuals, and regions usually, is ignored in the environmental economic literature. However, income inequality is a socioeconomic problem faced by both groups of countries, whether developed or developing countries (Uzar and Eyuboglu 2019). For example, Coondoo and Dinda (2008) have proven that income disparity has an important association on the emission level for different income groups such as Africa, America, Asia, and Europe through empirical analysis. Therefore, understanding the connection between income disparity and emission can guide the practices of politicians who aim to diminish inequality and prevent climate change (Rojas-Vallejos and Lastuka 2020). The growing inequality in income between rural and urban populations is a factor affecting the future economic growth and social stability of countries (Wolde-Rufael and Idowu 2017). Consequently, it can be expected that a fairer income dispersion will contribute to social accordance and contribute to the formation of a public opinion to improve environmental quality (Coondoo and Dinda 2008). Europe is a more equitable place to live than any other country in terms of income and resource distribution. However, the Gini coefficient shows that income inequality in the EU has increased over the last two decades. The increase in inequalities is more pronounced in the context of digitalization and technological acceleration (European Commission 2020). Piketty and Saez (2003) mentioned that inequality has increased with the new industrial revolution, but it will tend to decrease again with the benefit of innovations. In addition to this affirmative contribution to income inequality, technology innovation can lead to the development of more green energy and energy-saving products. The increase in technological innovations contributes to the improvement of environmental quality by reducing fossil energy consumption. Therefore, technological innovation emerges as an important variable that affects the environment through energy use (Tang and Tan, 2013). The EU performs strongly in terms of technological developments in various fields such as environment, climate, and energy. About 20% of the publication, total research and development, and patenting activity in the world belong to the EU. Therefore, the EU plays a significant role in terms of innovation and knowledge (European Commission 2020).

The effect of international trade on environmental degradation has recently become one of the issues that have fascinated attention (Hasanov et al. 2018). Since trade movements such as exports and imports, output, and energy use tend to move together, it is important to know the linkage between these variables for the growth and development of countries. Understanding the nexus between energy, output, and trade also contributes to the development of a new effective energy and environmental policy (Sadorsky 2012). International trade can affect CO_2_ emissions through increasing income, technology diffusion, and industry upgrading. While imports have the effect of reducing CO_2_ emissions by leading to the spread of technology, industry improvement, and production substitution, exports have the impact of increasing CO_2_ emissions through economic expansion, energy use, and increase in consumption (Wu et al. 2021). The new trade policy strategy of the EU was determined by the European Commission in 2021. In order to fully implement the UN Sustainable Development Goals, the EU’s trade policy is focused on supporting the green and digital transformation of the EU economy. The EU ranks first in the world as the largest trader of agricultural and manufactured services, goods, and investments (Rueda-Cantuche et al. 2021). According to the Eurostat database, while the EU’s goods export earnings were 1435.6 billion EUR in 2010, it reached 1932.2 billion EUR in 2020. In addition, the EU’s service export earnings, while 591.5 billion EUR in 2010, reached 867.1 billion EUR in 2020. The telecommunications, computer, and information services have a significant share in service exports with 20.1% in 2020. In line with these explanations, high-tech products have a significant share in the EU’s export earnings and this development shows the positive results of efforts toward improvement of environmental quality.

Recent developments for the EU demonstrate that energy source diversification continue, but fossil fuel consumption, which causes CO_2_ emissions, is still significant (Paraschiv and Paraschiv 2020). According to the BP Statistical Review of World Energy (2021) report, the CO_2_ emissions in the EU countries, which were 3386.4 million tons in 2010, decreased to 2550.9 million tons in 2020. The energy policies of the EU have an important place in this positive development in terms of environmental quality. According to the same report, renewable energy production in EU countries reached 710.4 terawatt hours (279.7 terawatt hours in 2010). In addition, the highest share (about 56%) in renewable energy generation belongs to wind energy in 2020. However, consumption of non-renewable energy sources is higher than renewable energy in EU countries. In 2020, while the non-renewable energy consumption is 55.74 EJ, the renewable energy consumption is 6.97 EJ for EU. In 2020, major components of fossil fuels were oil (35.9%), coal (10.6%), and natural gas (24.5%) in EU. The oil consumption in EU has decreased from 11 998 thousand barrels per day in 2010 to 9 774 thousand barrels per day in 2020. On the other hand, crude oil production in EU has decreased from 606 thousand of barrels per day in 2010 to 385 thousand of barrels per day in 2020. It is estimated that the interest of non-fossil fuels in primary energy in the EU will increase to 40% in 2030 through the European Green Deal (IEA 2020).

A significant international organization in the struggle opposite climate change is the EU. The European Commission has taken a decision to ensure carbon neutrality in the EU by 2050 (Duarte et al. 2021). To help achieve this goal, the EU’s new proposal is the hydrogen strategy (IEA 2020). Among the Sustainable Development Goals (SDG) of United Nations, a target has been set to reduce inequality by 2030 (Filauro 2018). Within the framework of SDG, in order to reduce the emission intensity of oil a gas production, resources with lower emission intensity are preferred. Thus, the global average emission density of gas and oil production is projected to decrease by approximately 40% over the period 2019–2030 (IEA 2020).

The EU faces challenges such as loss of biodiversity, climate change, population aging, and increasing inequalities (European Commission, 2020). Inequality in income deploy has the potential to bring about social, environmental, and economic issues (Uzar and Eyuboglu 2019). The increase in environmental problems and income inequality necessitated questioning whether environmental performance is affected by inequality. Also, there are significant differences in indicators such as income, energy efficiency, and emissions per capita in current EU member states (Padilla and Duro 2013). Furthermore, the nexus between exports of goods and services and pollution is among the topics on the agenda. In this paper, the consequences of innovations, exports, and income distribution on CO_2_ emissions were investigated with empirical analyses in order to determine the determinants of environmental pollution in the European Union.

It is known that world prosperity and sustainability are threatened by environmental degradation (Chu and Le 2022) and environmental degradation promotes climate change. Therefore, the link between structural changes, economic development, and environmental quality should be examined in order to reduce the impact of climate change and prevent future losses than can affect society and the economy (Ikram et al. 2021). For example, technological innovations affect some sectors and industries of the economy (Ślusarczyk 2018). The interaction between resource efficiency and technological innovations can positively affect environmental quality. Differences in environmental quality indicators in developed and developing countries require examining the factors affecting environmental quality. Additionally, there are inequalities in access to new technologies around the world. Adoption of technological innovations may also help explain a country’s level of environmental quality. Increasing concerns about environmental protection in the EU in recent years have brought new environmental technology applications to the agenda through environmental policies (Pociovălişteanu et al. 2015). Therefore, the EU is leading the creation of policies that support sustainable development by drawing attention to climate and environmental problems (Hedberg and Šipka 2022). There are studies in the literature that examine the EKC hypothesis in terms of income and environmental quality. However, the number of studies examining the impact of technological innovations on environmental quality, especially in the EU, is limited. Therefore, it is important to empirically determine the overall effects of technological innovations on environmental quality.

The present study can make the following contributions to the environmental literature. (1) The study may be significant in that it is the only one that deals with environmental quality in the EU in the light of different indicators such as exports, technological innovations, and income inequality. (2) The EKC hypothesis has a vital role in the analysis of the ecological effects of economic growth (Zhang and Zhao 2014). In this paper, the EKC hypothesis was used to explore the association between the variables determined to affect the environment based on the literature. Therefore, the role of dynamics adopted to explain emissions in the EKC hypothesis is tried to be determined. (3) Sustainable development has three pillars as social, economic, and environmental factors (Clement and Meunie 2010). Social choice can lead to a cleaner environment, as income distribution affects social preferences (Qu and Zhang 2011). While income inequality is observed in the development of rapid economic development, fairer income distribution guides the preservation of social stability, equality, and justice (Zhang and Zhao 2014). At this time, the association between inequality and environmental quality is also investigated in this study. (4) Globally, economic growth and population growth are driving rises in CO_2_ emissions from fossil fuels (Sohag et al. 2015). The difference in CO_2_ emission levels in the world is related to economic development, technological innovation, and resource equipment (Wu et al. 2021). In order to achieve low carbon emissions, it is necessary to identify better ways to reduce energy use and carbon emissions in the industry (Zhang et al. 2016). This study also examines the role of technology innovation on CO_2_. (5) Departing from the previous papers’ developing countries, this paper focuses on a panel of European countries’ developed countries. (6) Instead of traditional econometric models such as DOLS, FMOLS, PMG, vector error correction model (VECM), and/or vector autoregressive (VAR) framework, which are quite frequently used in the empirical literature, in this study, a new econometric method known as panel data vector autoregression (PVAR) is used. In the PVAR model, it allows the correct interaction to be revealed by allowing mutual interactions without making any exogenous and endogenous distinction between the variables.

The remainder of the study is arranged in the following way: the “Literature” section clarifies the literature on exports, technology, and income inequality toward environmental pollution. The model and data is presented in the “Model and data” section. The “Methodological explanations” section includes methodological explanations. Empirical findings are explained in the “Empirical findings and synthesis of results” section. In the last section, policy implications for the findings obtained from the analyses are suggested.

## Literature Review

With the world’s exposure to environmental problems such as climate change and global warming, researchers have focused on researching the factors affecting these issues since the 1990s (Halicioglu 2009) and began to study the association inequality/income and environmental degradation (Borghesi 2006). Differences in income distribution affect individuals’ attitudes toward the environment. In the case of more poverty, while the preferences of individuals are the struggle for survival rather than environmental degradation, the rise in the income level reveals that the preferences are directed to the need to protect the environment (Morse 2018).

In the literature part of the study, applied studies that deal with the factors affecting the environment are discussed in detail in two separate groups as single country and multi-country.

### Association between pollution and income inequality

The Gini coefficient was developed to measure income inequality. In other words, the Gini coefficient is used to forecast the influence of income inequality on the pollution (Heerink et al. 2001). A few of the studies dealing with this nexus belong to Bai et al. (2020), Grunewald et al. (2017), Zhu et al. (2018), and Clement and Meunie (2010). There are a number of studies examining the association between income disparity and environmental pollution. While some papers suggest that income improves environmental quality, the evidence for the detrimental effect of inequality should not be underestimated. An explanation for the rich preferring less environmental degradation than the poor stems from the EKC hypothesis (Drabo 2011). Boyce (1994) pioneered the theoretical investigation of how inequality affects environmental quality. According to the author, inequality negatively affects environmental quality. Based on the positive and negative effects of income disparity on the environmental degradation, Borghesi (2006) stated that lower income inequality reduces emissions due to the lower consumption levels of the poor compared to the rich. Conversely, lower inequality may also increase emissions, as the poor use energy less efficiently than the rich.

Baek and Gweisah (2013) is one of the only country papers examining the interrelation between income disparity and pollution. By applying the ARDL approach, the Baek and Gweisah (2013) determined that more equality in income dispersion would improve environmental property for the USA. Analyzing the association between environmental degradation and income disparity within the framework of the EKC, Demir et al. (2019) showed that the rise in income disparity in Turkey reduces pollution. Kusumawardani and Dewi (2020) examined the link between pollution, urbanization, and income inequality in Indonesia. By applying the ARDL test, it was determined that income disparity reduces pollution. The EKC hypothesis was also supported. Otherwise, Uzar and Eyuboglu (2019), who studied the association between environmental degradation and income inequality for Turkey, found that income disparity deteriorates environment. Also, the EKC theory is available for Turkey. Bai et al. (2020) tested environmental pollution and income inequality in China using panel fixed effect regression. The findings showed that income inequality hinders renewable energy technological innovations, and as a result, environmental pollution increases.

Multi-country papers examining the income inequality-pollution nexus are more numerous than a single country. For 35 countries with different income levels (12 middle-income, 11 low-income, and 12 high-income countries), Borghesi (2006) showed that greater income inequality reduces pollution, according to the pooled OLS model. Qu and Zhang (2011) demonstrated that a rise in income dispersion in 36 countries develops environmental quality by using panel data analysis. For 86 countries, Guo (2013) studied the linkage between income disparity and pollution using different techniques (fixed effect, random effect, and generalized least squares). The analysis findings are as follows: (1) The EKC hypothesis is valid in all of the country groups examined. (2) Income disparity has an unfavorable influence on pollution. Using panel smooth transition regression analysis, Rojas-Vallejos and Lastuka (2020) found that income disparity reduced carbon emissions in 68 countries between 1961 and 2010. Conversely, Clement and Meunie (2010) indicated that the EKC theory is valid for SO_2_ emissions for developing and transition countries. Also, an increase in equality affects water pollution in developing countries, although the Gini index does not effect SO_2_ emissions. Drabo (2011) has shown that income inequality deteriorates air quality by increasing air pollutants such as CO_2_ and SO_2_ and water pollution indicators in developed and developing countries. Golley and Meng (2012) examined the changes in CO_2_ emissions across households of different income levels in China, using a survey method, and the findings revealed that income inequality increases emissions. Zhang and Zhao (2014) studies the association between emissions and income inequality for different regions of China. The impact of income growth on pollution differed against areas of China. It has been observed that the influence of income disparity on pollution is greater in the eastern area compared to the western area. In addition, income inequality rises pollution for China. Grunewald et al. (2017) investigated the association between emission and income disparity by applying fixed effect regression analysis for 158 countries belonging to different income categories. The results of the paper revealed that income inequality reduces emission in middle- and low-income countries, but income disparity rises pollution in high- and middle-income countries. Kasuga and Takaya (2017) analyzed the influence of income disparity on environmental degradation for Japanese cities and the findings indicated that disparity negatively affects quality of air for commercial and residential buildings. Using DOLS and FMOLS estimators, Wolde-Rufael and Idowu (2017) determined that energy use and income are among the most important factors of pollution, but income disparity is the factor that affects emissions the least for India and China. Morse (2018) found that income distribution is related to the Environmental Performance Index (EPI) by using 16 environmental performance indicators in 180 countries. Zhu et al. (2018) investigated the influences of income disparity and urbanization on pollution for BRICS using panel quantile regression. The findings showed that income disparity has an affirmative influence on emission for high- and middle-emission countries. Also, the EKC theory was supported in the relevant economies. Chen et al. (2020a, b) tested the association between pollution and income dispersion within the structure of the EKC in G20. The findings of the quantile regression analysis showed that a fairer dispersion of income reduces emission for developing countries, while income disparity rises pollution for developed countries. Also, the EKC was supported for the G20. Sahu and Patnaik (2020) have obtained that income disparity increases emission for BRICS. By applying panel quantile approach, Chen and Lei (2018) showed that technological innovations reduce CO_2_ emissions more in countries with higher CO_2_ emissions than low-emission countries. On the other hand, Jorgenson et al. (2017) showed that income disparity has no effect on emission for the US states.

Papers analyzing the link between environmental degradation and income inequality are limited for the EU. Duarte et al. (2021) divided the EU countries into five income categories and investigated the link between environmental quality and income dispersion. The rise in income led to a rise in pollution for the low-income country group. Therefore, emissions would be reduced by reducing income inequality.

Based on income distribution and pollution literature, hypothesis 1 is as follows:

- Hypothesis 1. Income inequality has a positive influence on CO_2_ emissions.

### Association between pollution and exports

Exports have an important role in the calculation of national income (Bosupeng 2016). Exports can also contribute to environmental degradation. Higher export rise encourages environmental pollution with higher energy use.

Investigating the association between exports and CO_2_ emissions in China using VECM analysis, Michieka et al. (2013) found a Granger causality relationship from exports to emissions. Bosupeng (2016) studied the link between exports and emission using the Toda and Yamamoto causality test. A one-way causality relationship was found from CO_2_ emissions to exports in Austria, Canada, Bolivia, Ireland, Morocco, and Costa Rica. On the other hand, there are 12 countries where causality is detected from exports to pollution. Al-mulali and Sheau-Ting (2014) investigated the linkage between exports, imports, and pollution in 189 economies by applying the FMOLS estimator. The authors found an affirmative long-run nexus between pollution and trade variables. Mensah et al. (2019) studied the association GDP, fossil energy, oil prices, and pollution for 22 African countries. The results of the analysis made by dividing African countries into two groups as non-oil-exporting and oil-exporting economies revealed a one-way causality nexus between oil prices and carbon emissions. Also, oil prices rise carbon emission. Wu et al. (2021) determined that exports increase pollution in the conclusions of the GMM model. Contrary, analyzing the nexus between CO_2_ emissions, imports, and exports in for exporting economies, Hasanov et al. (2018) concluded that exports and imports reduce CO_2_ emissions. Mahmood et al. (2020) tested the nexus between exports, imports, and pollution in North African economies with fixed effect regression analysis. The EKC hypothesis was found to be valid for all countries. In addition, exports affect CO_2_ emissions negatively. Another study dealing with the impacts of international trade and urbanization on carbon emissions belongs to Muhammad et al. (2020). The findings have produced diverse results for countries according to income level. While exports increased pollution in low-middle-income economies, it reduced pollution in high- and low-income economies. In addition, the EKC hypothesis has been confirmed in high- and high-middle-income countries.

Based on the previous papers, we improve hypothesis 2 as follows:

- Hypothesis 2. Exports has a positive impact on CO_2_ emissions.

### Association between pollution and technological innovation

Innovation promotes products and activities that rise energy efficiency and consume less resources (Fernández et al. 2018). Technological innovation has taken its place in the environmental literature in order to ensure sustainable development, especially through energy. The environmental impact of innovation which is a significant input for economic growth is uncertain. While higher economic activity harms the environment by increasing energy usage, the innovation process can reduce pollution by reducing energy use (Fernández et al. 2018).

Samargandi (2017) found that technological innovation is insignificant in reducing CO_2_ emissions in Saudi Arabia. Chen et al. (2020a, b) found that innovation performance indicators have a major influence on emission from the transportation sector in China. Chien et al. (2021) analyzed the association between technological innovation and environmental degradation for Pakistan. While the EKC hypothesis was supported for Pakistan, the effect of technological innovation on emission was found to be unfavorable. Adebayo et al. (2021) used the non-linear ARDL technique and came to the conclusion that technological innovations do not affect CO_2_ emissions for Chile.

Irandoust (2016) examined nexus between R&D spending for technological innovation and CO_2_ emission for Nordic countries by constructing a VAR model. A causal linkage was found between technology innovation and pollution. Sinha et al. (2020a) tested the linkage between technological advances and degradation for Asia Pacific countries. The test results revealed that technological advances negatively affect environmental quality. In addition, a unidirectional causality nexus from technological advances to air quality has also been determined. By applying panel quantile regression analysis, Sinha et al. (2020b) found that technological progress increases air pollution in MENA countries. Wang et al. (2020a, b) tested the link between technology, renewable energy use, and pollution for N-11. Findings from various estimators such as AMG and CCEMG technology and renewable energy use are negatively associated with carbon emissions. Xie et al. (2021) researched the linkage between environmental quality and technology for 59 countries. According to the panel causality test results, technological progress has increased carbon emissions at different levels in countries. There are few papers that analyze the nexus between technology and pollution for the EU. For example, Fernández et al. (2018) showed that innovation reduces CO_2_ emissions in 15 EU countries using regression analysis. Testing the relationship between environmental degradation and technology in 19 EU countries with quantile regression analysis, Khan et al. (2021) have shown that technology contributes to mitigating carbon emissions.

Based on previous papers, we propose hypothesis 3 as follows:

- Hypothesis 3. Technological innovation has a negative impact on CO_2_ emissions.

### Association between pollution, exports, technology, and income distribution

There are also studies investigating the association between environmental pollution, exports, technology, and income distribution. However, it has been concluded that the previous literature has been judged to be rather limited.

Jiao et al. (2021) analyzed the link between oil prices, exports, income disparity, pollution, and technological innovations for India by applying the NARDL approach. While NARDL test results proved that technological innovations and exports rise CO_2_ emissions, long-term test results revealed that the rise in oil prices and income equality, as well as the decrease in exports, had an unfavorable influence on emissions. Salman et al. (2019) tested the influence of technological innovations, import, and export on pollution for ASEAN with panel quantile regression analysis. As a result, it has been determined that exports increase pollution, while technological innovations reduce pollution. Sharma et al. (2021) investigated the influence of technological advancement, export diversification, and income disparity on emission for BRICS countries. The long-term coefficients revealed that export diversification deteriorates air quality, but technological innovations improve quality of the environment. Wahab et al. (2021) tested the impacts of income, imports, exports, and technological innovations on environmental pollution in G7 countries using the CS-ARDL technique. The findings revealed that both exports and technological innovations adversely affect CO_2_ emissions.

Firstly, the literature has been presented by dividing income inequality-pollution, exports-pollution, technological innovation-pollution, and urbanization-pollution. Then, in previous studies, the interrelationship between pollution, income distribution, exports, urbanization, and technology in Table 1 and Table 2 is prepared separately as single country and multi-country.

**Table 1:** Literature on interrelationship between pollution, income distribution, exports, urbanization, and technological innovation for single country

Contributor(s)	Study period	Country	Methodology	Variables used	Relationship
Association between pollution and income inequality					
Baek and Gweisah (2013)	1967–2008	USA	ARDL	Income inequality, CO_2_ emission	GINI↑ CO_2_↑
Demir et al. (2019)	1963–2011	Turkey	ARDL	Income inequality, CO_2_ emission	GINI↑ CO_2_↓
Uzar and Eyuboglu (2019)	1984–2014	Turkey	ARDL	Income inequality, CO_2_ emission	GINI↑ CO_2_↑
Bai et al. (2020)	2000–2015	China	Fixed effect regression	Income inequality, CO_2_ emission	GINI↑ CO_2_↑
Kusumawardani and Dewi (2020)	1975–2017	Indonesia	ARDL	Income inequality, CO_2_ emission	GINI↑ CO_2_↓
Association between pollution and technological innovation					
Samargandi (2017)	1970–2014	Saudi Arabia	ARDL	Technology, CO_2_ emission	TI ≠ CO_2_
Adebayo et al. (2021)	1990–2018	Chile	Non-linear ARDL	Technological innovation, CO_2_ emission	TI ≠ CO_2_
Chien et al. (2021)	1980–2018	Pakistan	QARDL	Technology, CO_2_ emission	TI↑ CO_2_↓
Association between pollution and urbanization					
Chen et al. (2020a)	2003–2019	China	Benchmark regression	Urbanization, CO_2_ emission	URB↑ CO_2_↑
Zhang et al. (2021)	2008–2017	China	Panel regression	Urbanization, CO_2_ emission	URB↑ CO_2_↑
Association between income distribution, exports, technology, and CO_2_ emissions					
Jiao et al. (2021)	1980–2018	India	NARDL	Exports, income disparity, technological innovation, CO_2_ emission	EXP↑ CO_2_↑ GINI↑ CO_2_↑ or EXP↓ CO_2_↓
					GINI↑ CO_2_↓ (long run)

↑ and ↓ represent, respectively, increasing and decreasing, whereas ≠ presents no effect. Emission of carbon, exports, income disparity, technological innovation, and urbanization are indicated CO_2_, EXP, GINI, TI, and URB in order

**Table 2:** Literature on interrelationship between pollution, income distribution, exports, urbanization, and technological innovation for multi-country

Contributor(s)	Study period	Country	Methodology	Variables used	Relationship
Association between pollution and income inequality					
Clement and Meunie (2010)	1988–2003	Developing and transition countries	Fixed effect and GMM estimator	Income inequality, CO_2_ emission	GINI ≠ SO_2_
Guo (2013)	1980–2006	86 countries	Fixed and random effect, FGLS estimator	Income inequality, CO_2_ emission	GINI↑ CO_2_↓
Zhang and Zhao (2014)	1995–2010	China’s regions	Panel estimator	Income inequality, CO_2_ emission	GINI↑ CO_2_↑
Grunewald et al. (2017)	1980–2008	158 economies	Fixed effect estimator	Income inequality, CO_2_ emission	GINI↑ CO_2_↓ GINI↑ CO_2_↑
Jorgenson et al. (2017)	1997–2012	50 US states	Fixed and random effect estimator	Income inequality, CO_2_ emission	GINI ≠ CO_2_
Kasuga and Takaya (2017)	1990–2012	Japanese towns	Estimation of GMM	Income inequality, CO_2_ emission	GINI↑ CO_2_↓
Morse (2018)	1995–2013	180 countries	Panel regression analysis	Income inequality, Environmental Performance Index (EPI)	GINI↑ EPI↑
	1990–2014	G7 countries	Pooled mean group (PMG)	Income inequality, air pollution	GINI↑ air pollution↑
Rojas-Vallejos and Lastuka (2020)	1961–2010	68 countries	Panel smooth transition regression	Income inequality, CO_2_ emission	GINI↑ CO_2_↓
Chen et al. (2020a, b)	1988–2015	G20 countries	Quantile regression	Income inequality, CO_2_ emission	GINI↑ CO_2_↑
Kusumawardani and Dewi (2020)	1975–2017	BRICS countries	Robust panel data estimation methods	Income inequality, CO_2_ emission	GINI↑ CO_2_↓
Sahu and Patnaik (2020)	1991–2018	BRICS countries	Fixed effect estimator	Income inequality, CO_2_ emission	GINI↑ CO_2_↑
Duarte et al. (2021)	1999–2015	EU countries	Structural decomposition analysis (SDA)	Income inequality, CO_2_ emission	GINI↑ CO_2_↑
Association between pollution and exports					
Al-mulali and Sheau-Ting (2014)	1990–2011	189 countries	FMOLS estimation	Export, import, CO_2_ emission	EXP↑ CO_2_↑
Hasanov et al. (2018)	1995–2013	9 oil-exporting countries	PMG estimator	Exports, imports, CO_2_ emission	EXP↑ CO_2_↓
Mahmood et al. (2020)	1990–2014	5 North African countries	Fixed effect regression	Exports, imports, CO_2_ emission	EXP↑ CO_2_↓
Muhammad et al. (2020)	2000–2016	65 Belt and Road Initiative	2-stage least square	Export, CO_2_ emission	EXP↑ CO_2_↓ EXP↑ CO_2_↑
Wu et al. (2021)	2002–2017	Belt and Road Initiative	GMM	Exports, imports, CO_2_ emission	EXP↑ CO_2_↑
Association between pollution and technological innovation					
Chen and Lei (2018)	1980–2014	30 countries	Panel quantile regression	Technology, CO_2_ emission	TI↑ CO_2_↓
Sinha et al. (2020b)	1990–2017	MENA	Quantile-on-quantile	Technology index, air pollution	TI↑ air pollution↑
Khan et al. (2021)	1995–2019	19 European Union	Quantile regression	Technology, CO_2_ emission	TI↑ CO_2_↓
Wang et al. (2020a, b)	1990–2017	N-11 countries	CCEMG and AMG estimator	Technology, CO_2_ emission	TI↑ CO_2_↓
Xie et al. (2021)	1998–2016	59 countries	Panel quantile regression	Technology, CO_2_ emission	TI↑ CO_2_↑
Association between pollution and urbanization					
Ali et al. (2019)	1972–2014	Emerging economy	ARDL	Urbanization, CO_2_ emission	URB↑ CO_2_↑
Kasman and Duman (2015)	1992–2010	EU member	Panel cointegration, panel causality test	Urbanization, economic growth, trade, CO_2_ emission	URB↑ CO_2_↑
Lee and Zhao (2023)	2000–2020	96 countries	Finite mixture model	Urbanization, foreign direct investment, CO_2_ emission	URB↑ CO_2_↑
Nuţă et al. (2023)	1995–2019	European and Asian emerging economies	FGLS	Urbanization, economic growth, renewable energy, CO_2_ emission	URB↑ CO_2_↑
Sikder et al. (2022)	1995–2018	Developing economics	ARDL	Urbanization, economic growth, energy, CO_2_ emission	URB↑ CO_2_↑
Wang et al. (2018)	1980–2011	170 countries	Panel cointegration	Urbanization, economic growth, energy use, CO_2_ emission	URB↑ CO_2_↑
Association between exports, technology, income distribution, and CO_2_ emissions					
Sinha et al. (2020a)	1990–2017	Asia Pacific economies	Approach of quantile	Technology index, air pollution	TI↑ air pollution↑
Sharma et al. (2021)	1990–2018	BRICS countries	CS-ARDL	Export diversification, technology innovation, income inequality, CO_2_ emission	TI↑ CO_2_↓ EXP↑ CO_2_↑
Salman et al. (2019)	1990–2017	ASEAN economies	Regression of quantile	Exports, imports, technology innovation, CO_2_ emission	TI↑ CO_2_↓ EXP↑ CO_2_↑
Wahab et al. (2021)	1996–2017	G7 countries	CS-ARDL	Exports, imports, technology innovation, CO_2_ emission	TI↑ CO_2_↓ EXP↑ CO_2_↓

↑ and ↓ represent, respectively, increasing and decreasing, whereas ≠ presents no effect. Emission of carbon, emission of sulfur oxide, exports, income disparity, technological innovation, urbanization, environmental performance index, are indicated CO_2_, SO_2_, EXP, GINI, TI, URB, EPI in order

This paper theoretically makes a contribution to the current environment economic literature in several aspects: first, from the previous papers on developing countries, this paper focuses on EU countries; second, we explore the role of “income inequality” and “technology innovation” on pollution.

## Model and data

This paper analyzes the relationship between income inequality, technological innovations, export, and environmental pollution by including the EKC hypothesis for the period from 2005 to 2019 in European Union countries (EU-27^1^). The main function describing this relationship is constructed as follows, inspired by Jiao et al. (2021), Wahab et al. (2021), and Sharma et al. (2021):

1 $${\text{CO}}2=f({\text{GDP}},\mathrm{ GDP}2,\mathrm{ TEC},\mathrm{ GINI},\mathrm{ EXP},\mathrm{ URB})$$

From this function, a basic panel data model can be written as follows:

2 $${{{\text{CO}}}_{2}}_{it}={\alpha }_{0}+{\beta }_{1}{{\text{GDP}}}_{it}+{\beta }_{2}{{\text{GDP}}2}_{it}+{\beta }_{3}{{\text{TEC}}}_{it}+{\beta }_{4}{{\text{GINI}}}_{it}+{\beta }_{5}{{\text{EXP}}}_{it}+{\beta }_{6}{{\text{URB}}}_{it}+{\varepsilon }_{it}$$

where CO_2_, GDP, GDP2, TEC, GINI, EXP, and URB represent CO_2_ emissions, economic growth, squared of economic growth variable, technological innovations, Gini index, export, and urbanization, respectively. Variable definitions and data sources are presented in Table 3. *i* denotes number of units and *t* implies time period. Also, $\alpha$ and *ε* imply constant term and error term, respectively. Logarithmic transformation is applied to the data set to alleviate the fluctuations in the data and to reach more robust findings empirically.

**Table 3:** Data definitions and sources

Abbreviation	Definition	Source
CO_2_	CO_2_ emissions (metric tons per capita)	WDI
GDP	GDP per capita (constant 2015 US$)	WDI
GDP2	Squared of GDP	WDI
TEC	Patent applications, residents	WDI
GINI	Gini coefficient of equivalized disposable income	Eurostat
EXP	Exports of goods and services (constant 2015 US$)	WDI
URB	Urban population (% of total population)	WDI

## Methodological explanations

IPS (CIPS) by Pesaran (2007) is employed as follows:

3 $${\text{CIPS}}(N,T)={N}^{-1}{\sum }_{\dot{{\text{I}}}=1}^{N}{t}_{i}(N,T)$$

where $C{\text{IPS}}\left(N,T\right)$ is the cross-section augmented form of the IPS unit root test developed by Im et al. (2003) and *t*_*i*_ $\left(N,T\right)$ is the cross-section augmented Dickey-Fuller statistic.

In this study, the relationships between pollution, growth, technological innovations, income inequality, exports, and urbanization are investigated by adopting the panel vector autoregression (VAR) approach as follows (Abrigo and Love 2016):

4 $${Y}_{it}={Y}_{it-1}{A}_{1}+{Y}_{it-2}{A}_{2}+\dots +{Y}_{it-p}{A}_{p-1}+{Y}_{it-p}{A}_{P}+{X}_{it}B+{u}_{it}+{e}_{it}$$

where ${Y}_{it}$ is $\left(kxk\right)$ vector of explanatory variables, ${X}_{it}$ is $\left(1xl\right)$ vector of exogenous covariates, and ${u}_{i}$ and ${e}_{it}$ are vectors of dependent variable-specific panel fixed effects and idiosyncratic errors, respectively.

Panel VAR models have some advantages when compared to alternative model structures: The most important advantage is that it allows examining all variables internally. In addition, since the lagged values of all variables are also considered in the model, it has a non-static structure (Canova and Ciccarelli 2013). The interdependencies and cross-sectional heterogeneities in the panel were pointed out by Abrigo and Love (2015). Heterogeneity between units is captured by the fixed effect variable. This is the situation where the ordinary least squares (OLS) method cannot be applied. At this point, the error term in dynamic panels is needed. This situation is eliminated with the panel VAR GMM style estimation method. Thus, the PVAR approach overcomes endogeneity among variables and heterogeneity within the panel. This method deals with short- and long-run dynamic relationship in detail with long-run coefficient estimation, short-run impulse-response graphs, and variance decomposition analysis.

## Empirical findings and synthesis of results

In this study, first of all, CIPS stationarity approach, which is a second-generation panel unit root test, is applied. The results of this test, whose most important advantage is to consider the cross-sectional dependence of the panel, are presented in Table 4. The stationarity of each series is analyzed for the two models with intercept and intercept and trend. The results mean that CO_2_, GINI, and URB series are stationary at level in both models, while other variables are stationary at first difference. Since the test results illustrate that none of the series has the integration degree of I(2), it reflects that it is possible to proceed to the next step and estimate to long-run coefficients.

**Table 4:** Cross-sectional dependency and unit root test

CSD test	Stat		Prob	
Pesaran (2004)	62.540		0.000	
Variables	Intercept		Intercept and trend	
	Level	1st difference	Level	1st difference
CO_2_	− 2.578^***^	− 4.349^***^	− 2.743^**^	− 4.107^***^
EXP	− 1.746	− 5.221^***^	− 1.708	− 5.632^***^
TEC	− 1.791	− 5.453^***^	− 2.518	− 5.568^***^
GINI	− 2.106^*^	− 4.947^***^	− 2.771^**^	− 4.648^***^
URB	− 2.835^***^	− 3.395^***^	− 1.192^***^	− 4.899^***^
GDP	− 1.655	− 5.051^***^	− 2.017	− 5.969^***^
GDP^2^	− 1.631	− 5.084^***^	− 2.013	− 5.991^***^

^*^, ^**^, and ^***^ mean 10%, 5%, and 1% statistically significance level, respectively

In this study, panel vector autoregression (PVAR) approach is adopted to estimate the long- and short-run relationships between series. Primarily, it is essential to determine the optimal lag length for the PVAR approach to be applied (Table 5). Since the smallest values in two of these criteria (MBIC and MQIC) are in the first lag length, lag 1 is taken as the basis in this study.

**Table 5:** Panel VAR lag order selection

Lag	CD	*J*	*J*-value	MBIC	MAIC	MQIC
1	1	224.309	0.0000417	− 598.659	− 69.69096	− 282.1015
2	1	116.8127	0.0945566	− 431.8327	− 79.18732	− 220.7943
3	− 132.7385	51.13722	0.3897393	− 223.1855	− 46.86278	− 117.6663

The first striking result is the existence of a U-shaped relationship between economic growth and pollution in EU-27 countries. This relationship can be understood from the fact that the GDP2 variable has a positive effect, despite the negative effect of the GDP on pollution. This result is related to the fact that development in the direction of economic growth in these countries cause significant environmental costs in the long run. According to another of the main findings of the study, an increase in patent applications used as a proxy for technological innovations mitigates environmental pollution by about 0.02 percent in these countries. This result is in line with Wang et al. (2020a, b), Fernández et al. (2018), Khan et al. (2021), Salman et al. (2019), Sharma et al. (2021), and Wahab et al. (2021). This finding confirms that technological developments in European Union also serve environmental quality. Considering that technological innovations are also an important component for growth, it is expected that the transfer of resources to technological developments will both contribute to growth and reduce pollution in these countries in the long run. Moreover, it is possible to reduce the environmental cost of the growth achieved due to such environmentally friendly technological developments. However, an increase in technological innovations is observed to reduce economic growth by about 0.002 percent in these countries (Table 6). Even if this is considered a slight effect, it can be associated with the costs of technological developments. Also, there is a U-shaped relationship between economic growth and technological innovations in the long run. Therefore, it can be said that the income increase in EU countries is used in unproductive areas first, but after the income exceeds a certain level, it is used in more productive areas. All these results draw attention to the growth-technology-pollution link and emphasize the versatility of this link.

**Table 6:** The long-run estimates

	CO_2_	GDP	GDP2	TEC	GINI	EXP	URB
L.CO_2_	− 0.013	0.003	0.048	− 9.988	− 0.045	− 0.006	− 0.000
L.GDP	− 12.213^***^	− 5.192^***^	− 39.443^***^	− 65.176^***^	− 40.375^***^	− 13.270^***^	− 0.051^***^
L.GDP2	1.357^***^	0.600^***^	4.567^***^	7.682^***^	4.652^***^	1.523^***^	0.005^***^
L.TEC	− 0.021^***^	− 0.002^***^	− 0.021^***^	− 0.182^***^	− 0.041^***^	− 0.008^***^	− 0.000^***^
L.GINI	0.034^***^	0.108^***^	0.840^***^	1.723^***^	0.725^***^	0.246^***^	− 0.000^**^
L.EXP	0.094^***^	0.019^*^	0.158^*^	0.751^***^	0.244^**^	0.052^*^	0.000
L.URB	2.336^***^	− 1.949^***^	− 15.520^***^	− 42.902^***^	− 16.498^***^	− 3.796^***^	0.017^***^

^*^, ^**^, and ^***^ mean 10%, 5%, and 1% statistically significance level, respectively

The findings of the relationship between income inequality and pollution for the EU-27 imply that an increase in income inequality in these countries increases emissions by about 0.03 percent. A similar finding compared to the relevant literature is also encountered in Drabo (2011), Baek and Gweisah (2013), Golley and Meng (2012), Zhang and Zhou (2014), Uzar and Eyuboglu (2019), Bai et al. (2020), Chen et al. (2020a, b), and Duarte et al. (2021). This result reflects that income inequality not only has economic and social dimensions but also that its environmental effects are inevitable. This result can be associated with the power of high-income groups in economic and political processes in case of income inequality. This means that individual and/or firms engaged in production and consumption activities that degrade environmental quality gain power with the increase in inequality and in this case, environmental policies cannot be implemented. Accordingly, while economic growth first has a reducing effect on income inequality, inequality increases after the income level of the countries exceeds a certain level. That is, the increase in economic growth results in a certain segment of the society gaining power over time and taking a larger share of the pie. The long-run effect of increasing inequality on economic growth, technological innovations, and exports can be interpreted because of this. According to the coefficient estimation results, an increase in Gini index increases economic growth by about 0.11 percent, technological innovations by about 0.75 percent, and exports by about 0.24 percent. Therefore, as a result of inequality, those who hold a large part of the income contribute to the production, technological investment, and trade processes with their economic and political power and their struggle to maintain this power.

Another finding is that export has a negative impact on environmental quality (as seen in Wu et al. 2021; Muhammad et al. 2020; Jiao et al. 2021; and Salman et al. 2019). In other words, an increase in exports in EU-27 countries increases carbon emissions by about 0.09 percent. This result reflects the high share of polluting sectors in exports in these countries. In addition, while exports contribute to economic growth and technological innovations, it causes income inequality. Therefore, although the economic contributions of exports are observed in these countries, the environmental and social costs are inevitable. Based on this result, although it is not possible for the economic contributions of exports to eliminate environmental costs for the period under consideration, it can be considered that this development may be achieved with appropriate measures for the future.

In addition to the Gini index and exports, the results confirmed that urbanization is another factor that causes pollution. Accordingly, an increase in urbanization increases CO_2_ emissions by about 2.33 percent. In addition, urbanization has a negative effect on economic growth, technological innovations, and exports. Therefore, urbanization harms sustainable development in these countries. Due to the increase in urban population density caused by urbanization, infrastructure problems result in the transfer of resources to such areas. This problem, which arises with urbanization, slows down the pace of technological innovations that feed economic activities in the long run, causing a decline in production, growth, and trade.

Considering that the main hypotheses of the study suggest a negative relationship between technological innovations and emissions and a positive relationship between exports and income inequality and emissions, it is observed that all three hypotheses are confirmed by the long-run coefficient estimation results. On the other hand, it is a fact that the detrimental effect of export and income inequality on environmental quality is greater than the pollution-reducing effect of innovations.

Other columns in the table give clues about the long-run dynamic relationships between variables. Accordingly, while income inequality and exports have a positive effect in growth, technological innovations and urbanization negatively affect growth. This result may be interpreted as income distribution being more in favor of segments that have an important role in economic growth. In the model where, technological innovations are the dependent variable, there is a U-shaped relationship between economic growth and technological innovations. Moreover, while income inequality and exports have a positive effect, urbanization has a negative effect on technological innovations. Additionally, it is clear that there is a U-shaped relationship between economic growth and GINI and export and urbanization. Technological innovations and urbanization have an improving effect on income distribution in these countries. However, it is a fact that exports cause income inequality. Another result is that technological innovations have a negative bur insignificant effect on exports, while the increase in income inequality makes a positive contribution.

**Fig. 1:**
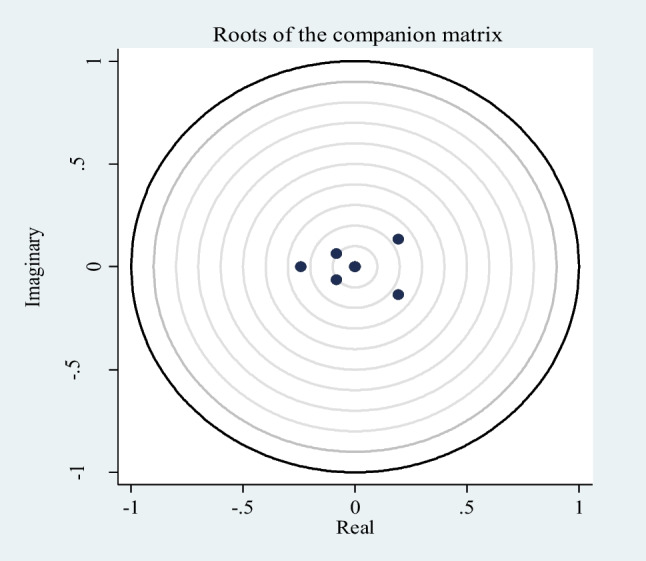
Stability of PVAR model

It is understood that all eigenvalues and modules are less than 1 in Table 7. In this case, it is clear that the stability condition of the model is provided (Fig. 1). Fulfilling this condition also makes it possible to perform other tests that the PVAR method allows. These are causality test, variance decomposition analysis, and obtaining impulse-response functions.

**Table 7:** Eigenvalue stability condition

Eigenvalue		Modulus
Real	Imaginary	
− 0.242	0	0.242
0.193	− 0.135	0.236
0.193	0.135	0.236
− 0.084	− 0.063	0.105
− 0.084	− 0.063	0.105
− 0.002	0	0.002
− 0.000	0	0.000

When Table 8, which gives an idea about the causality relationship between the series, is examined, first of all, it draws attention that there is a unidirectional causality relationship from all explanatory variables to emissions. This result implies that economic growth, technological innovations, income inequality, exports, and urbanization are powerful dynamics that explain environmental degradation. Therefore, it can be said that this causality relationship also supports the long-run coefficient estimation results. Also, there is a bidirectional causality between GDP and technological innovations, GDP and Gini index, GDP and exports, and GDP and urbanization. In addition, there is bidirectional causality between technological innovations and Gini index, technological innovations and exports, and technological innovations and urbanization. Moreover, another bidirectional causality relationship is detected between Gini index and both exports and urbanization.

**Table 8:** Granger causality Wald test results

	CO_2_	GDP	GDP2	TEC	GINI	EXP	URB
CO_2_		0.101	0.330	0.000	0.287	0.076	1.634
GDP	157.448^***^		712.479^***^	596.486^***^	1542.100^***^	811.416^***^	117.814^***^
GDP2	139.993^***^	729.210^***^		609.198^***^	1399.739^***^	735.782^***^	105.991^***^
TEC	13.207^***^	19.441^***^	17.568^***^		67.974^***^	25.213^***^	16.850^***^
GINI	14.369^***^	485.019^***^	467.885^***^	395.601^***^		522.308^***^	4.803^**^
EXP	7.584^***^	2.746^*^	2.815^*^	9.930^***^	5.653^***^		1.027
URB	100.292^***^	151.869^***^	153.585^***^	151.789^***^	141.982^***^	148.787^***^	

^*^, ^**^, and ^***^ mean 10%, 5%, and 1% statistically significance level, respectively

The variance decomposition analysis is provided in Table 9. Variance decomposition shows the percentage of variation in one variable by the accumulated shock in another variable over time. The main purpose of variance decomposition analysis is to divide the total variance in a response variable into the components included in the model. The first part of the table contains the response of CO_2_ emissions to all variables in the model. Accordingly, about 44 percent of the changes in the variance of CO_2_ over a 10-year period are clarified by changes in itself and about 45 percent by changes in GDP. The most important interpretation of these results is that the main source of environmental pollution in the next 10-year period will be economic growth. Therefore, the necessity of a formula that guarantees sustainable growth is once again emphasized. This formula should include the spread of such technology patents in connection with a production structure that supports green technologies. In addition, an environmentally friendly export structure and sustainable cities should also contain policies. About 90 percent of the changes in GDP are explained by changes in itself, while about 9 percent of the changes in GDP are explained by technological innovations. In addition, about 68 percent of the changes in the variance of technological innovations, about 75 percent of the changes in the variance of the Gini index, and about 84 percent of the changes in the variance of exports are explained by changes in GDP. While about 45 percent of the changes in the variance of the urbanization variable are due to changes in itself, about 33 percent are explained by the changes in GDP.

**Table 9:** Variance decomposition results

Response variable and Forecast horizon (years)	Impulse						
	CO_2_	GDP	GDP^2^	EXP	TEC	GINI	URB
CO_2_							
0	0	0	0	0	0	0	0
1	1	0	0	0	0	0	0
2	0.4817918	0.4676287	0.0398621	0.0074966	0.0026841	0.0003713	0.0001655
3	0.4434417	0.4591656	0.0352596	0.0067749	0.0546482	0.000364	0.0003461
4	0.4421879	0.4599378	0.0351833	0.006801	0.055112	0.0003814	0.0003967
5	0.4421315	0.459896	0.0351839	0.0067999	0.0552091	0.0003818	0.0003977
6	0.4421315	0.4598954	0.0351841	0.0068002	0.0552091	0.0003818	0.0003978
7	0.4421315	0.4598955	0.0351841	0.0068002	0.0552091	0.0003818	0.0003978
8	0.4421315	0.4598955	0.0351841	0.0068002	0.0552091	0.0003818	0.0003978
9	0.4421315	0.4598955	0.0351841	0.0068002	0.0552091	0.0003818	0.0003978
10	0.4421315	0.4598955	0.0351841	0.0068002	0.0552091	0.0003818	0.0003978
GDP							
0	0	0	0	0	0	0	0
1	0.0036472	0.9963528	0	0	0	0	0
2	0.0260487	0.8983395	0.0000399	0.0007047	0.0744339	0.000056	0.0003774
3	0.0261129	0.8959186	0.0001832	0.0008027	0.0764194	0.000096	0.0004672
4	0.0261179	0.8958277	0.0001958	0.0008027	0.0764898	0.000097	0.0004692
5	0.0261185	0.8958263	0.0001962	0.000803	0.0764896	0.000097	0.0004692
6	0.0261186	0.8958263	0.0001963	0.000803	0.0764897	0.000097	0.0004692
7	0.0261186	0.8958262	0.0001963	0.000803	0.0764897	0.000097	0.0004692
8	0.0261186	0.8958262	0.0001963	0.000803	0.0764897	0.000097	0.0004692
9	0.0261186	0.8958262	0.0001963	0.000803	0.0764897	0.000097	0.0004692
10	0.0261186	0.8958262	0.0001963	0.000803	0.0764897	0.000097	0.0004692
TEC							
0	0	0	0	0	0	0	0
1	0.0681529	0.7064793	0.0231216	0.2022462	0	0	0
2	0.0665208	0.692632	0.0195177	0.1716629	0.0489952	0.0002021	0.0004694
3	0.066957	0.6900122	0.019501	0.1701636	0.0526137	0.0002194	0.000533
4	0.0669501	0.6899948	0.0195059	0.1701519	0.0526398	0.0002212	0.0005363
5	0.0669501	0.6899928	0.0195066	0.1701513	0.0526417	0.0002213	0.0005363
6	0.0669502	0.6899926	0.0195066	0.1701512	0.0526419	0.0002213	0.0005363
7	0.0669502	0.6899926	0.0195066	0.1701512	0.0526419	0.0002213	0.0005363
8	0.0669502	0.6899926	0.0195066	0.1701512	0.0526419	0.0002213	0.0005363
9	0.0669502	0.6899926	0.0195066	0.1701512	0.0526419	0.0002213	0.0005363
10	0.0669502	0.6899926	0.0195066	0.1701512	0.0526419	0.0002213	0.0005363
GINI							
0	0	0	0	0	0	0	0
1	0.0304961	0.8072007	0.0427258	0.0033998	0.1161775	0	0
2	0.0445802	0.7531219	0.0391926	0.003024	0.1594882	0.0001403	0.0004527
3	0.0445306	0.7531618	0.039166	0.0030668	0.1594253	0.0001564	0.0004932
4	0.0445426	0.753091	0.0391688	0.0030667	0.159481	0.0001566	0.0004935
5	0.0445438	0.753089	0.0391687	0.003067	0.1594814	0.0001566	0.0004935
6	0.0445438	0.753089	0.0391687	0.003067	0.1594814	0.0001566	0.0004935
7	0.0445438	0.753089	0.0391687	0.003067	0.1594814	0.0001566	0.0004935
8	0.0445438	0.753089	0.0391687	0.003067	0.1594814	0.0001566	0.0004935
9	0.0445438	0.753089	0.0391687	0.003067	0.1594814	0.0001566	0.0004935
10	0.0445438	0.753089	0.0391687	0.003067	0.1594814	0.0001566	0.0004935
EXP							
0	0	0	0	0	0	0	0
1	0.0008548	0.9507501	0.0082938	0.0007554	0.002034	0.0373118	0
2	0.0278207	0.8445339	0.0077815	0.000977	0.0876273	0.0309391	0.0003206
3	0.0277799	0.8441348	0.0078486	0.0011196	0.0878179	0.0309117	0.0003875
4	0.0277915	0.8440585	0.0078574	0.0011201	0.0878756	0.0309091	0.0003879
5	0.0277935	0.8440545	0.0078575	0.0011206	0.0878771	0.0309089	0.0003879
6	0.0277935	0.8440545	0.0078575	0.0011206	0.0878771	0.0309089	0.0003879
7	0.0277935	0.8440545	0.0078575	0.0011206	0.0878771	0.0309089	0.0003879
8	0.0277935	0.8440545	0.0078575	0.0011206	0.0878771	0.0309089	0.0003879
9	0.0277935	0.8440545	0.0078575	0.0011206	0.0878771	0.0309089	0.0003879
10	0.0277935	0.8440545	0.0078575	0.0011206	0.0878771	0.0309089	0.0003879
URB							
0	0	0	0	0	0	0	0
1	0.2362368	0.0607024	0.0019534	0.0088826	0.0018173	0.0000105	0.690397
2	0.1648039	0.3260211	0.0185464	0.0088923	0.0015053	0.0000617	0.4801693
3	0.164548	0.3284142	0.0175597	0.0083822	0.0314661	0.0000888	0.449541
4	0.1643583	0.3288927	0.0175554	0.0084023	0.0316459	0.0000988	0.4490466
5	0.1643568	0.3288799	0.0175578	0.0084018	0.0316913	0.000099	0.4490135
6	0.164357	0.3288797	0.0175578	0.008402	0.0316914	0.000099	0.4490131
7	0.164357	0.3288797	0.0175578	0.008402	0.0316914	0.000099	0.449013
8	0.164357	0.3288797	0.0175578	0.008402	0.0316914	0.000099	0.449013
9	0.164357	0.3288797	0.0175578	0.008402	0.0316914	0.000099	0.449013
10	0.164357	0.3288797	0.0175578	0.008402	0.0316914	0.000099	0.449013

**Fig. 2:**
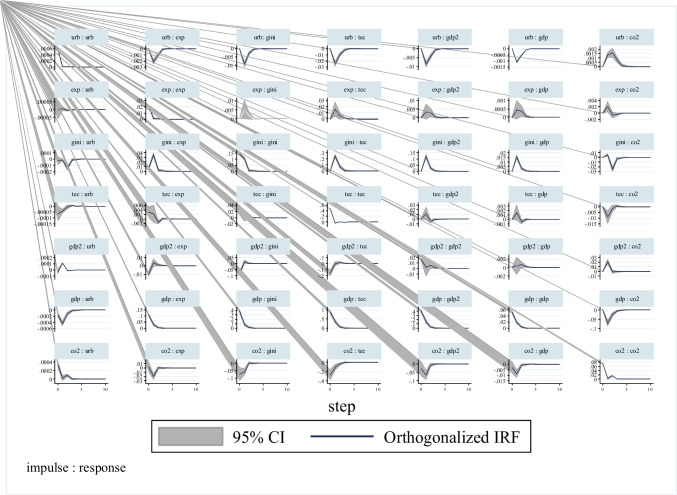
Impulse-response graphs

Impulse-response graphs are presented in Fig. 2. Impulse-response graphs illustrate the response of the dependent variable to a standard deviation shock in the explanatory variables. When Fig. 2 is examined, it is seen that a standard deviation shock in GDP first reduces emissions and then increases it. This result, which gives information about the short-run dynamics, is compatible with the U-shaped relationship between growth and pollution in the long run. As a matter of fact, standard deviation shock in GDP2 which first increases and then decreases emissions is another proof of this. The standard deviation shocks in the Gini index and technological innovations first reduce pollution and then increase it. However, a standard deviation shock in urbanization and exports first increases pollution and then decreases it. With these impulse-response functions obtained, it is clearly observed that there is a dynamic interaction between the explanatory variables in the model and the shocks in the dependent variable, especially in the base model where CO_2_ emission is the dependent variable.

## Conclusion and policy implications

Using panel data from 2005 to 2019, this study explored the nexus among economic growth, technological innovations, income inequality, exports, and CO_2_ emissions in EU-27 countries with consideration for dynamic interaction within the panel VAR method. Main results suggest that income inequality, exports, and urbanization have a positive impact on CO_2_ emissions, but technological innovations reduce emissions. Also, there is U-shaped relationship between economic growth and pollution in EU-27 countries meaning that EKC is invalid in the long run. Therefore, the interaction of all explanatory variables with pollution is determined in the model in which CO_2_ is dependent variable, which is adopted as the main focus of the study. Undoubtedly, the results from the causality analysis support this view. Accordingly, a unidirectional causality is found from all explanatory variables to CO_2_ emissions.

The findings of the study provide insight into the policy-making processes of EU-27 countries from various perspectives:

- First, deviations from long-run sustainable growth targets in these countries are evident from the U-formed relationship between GDP and CO_2_. According to this result, spreading the use of environmentally friendly energy in production processes and focusing on environmentally friendly technology investments should be primary priorities. In addition, a part of the income should be allocated to environmental regulations and policies, and the establishment of facilities with sustainable production processes should be encouraged.
- Second, the fact that income inequality is an element of the increase in pollution implies that social and economic power balances deteriorate as inequality increases in these countries. This imbalance leads to deterioration in resource allocation and causes countries to neglect environmental policies. Therefore, fair distribution of income rather than targeting improvement in growth performance alone is very critical in terms of environmental quality. In addition to the policy implementations aimed at ensuring fairness in income distribution in these countries, the production and consumption processes that harm the environment of the people who receive a high share of income should be limited with sanctions.
- Third, considering the pollution-reducing effect of technological innovations, policies that encourage the use of new environmentally friendly technologies should be adopted. Therefore, it may be possible to eliminate to some extent the environmental degradation caused by growth in the long run. In this process, incentives should be implemented to alleviate the cost burden, especially in the use of these new technologies by manufacturers. Moreover, this positive effect of technological innovations on the environment is associated with human capital investments. On other words, up-to-date educational infrastructure should be established and current conditions should be improved in order to raise qualified individuals with environmental awareness and high technology development skills.
- Fourth, the results related to exports imply that the polluting industries have a large share in trade. Also, it is related that the increase in exports means more domestic production and that these production processes deviate from the sustainable principle. In this case, investment and subsidy programs for clean sectors should be developed.

## Data Availability

The data that support the findings of this study are openly available on request.

## Footnotes

1 These countries are Austria, Belgium, Bulgaria, Croatia, Cyprus, Czech Republic, Denmark, Estonia, Finland, France, Germany, Greece, Hungary, Ireland, Italy, Latvia, Lithuania, Luxembourg, Malta, Netherlands, Poland, Portugal, Romania, Slovakia, Slovenia, Spain, and Sweden.
